# The Metabolic Score for Insulin Resistance (METS-IR), a Predictor of Cardiovascular Events, Relates to Disease Activity in Patients with Rheumatoid Arthritis

**DOI:** 10.3390/diagnostics15070861

**Published:** 2025-03-28

**Authors:** Antonio Aznar-Esquivel, Fuensanta Gómez-Bernal, María García-González, Marta Hernández-Diaz, Elena Heras-Recuero, Antonia de Vera-González, Alejandra González-Delgado, Adrián Quevedo-Rodríguez, Juan C. Quevedo-Abeledo, Santos Castañeda, Miguel Á. González-Gay, Iván Ferraz-Amaro

**Affiliations:** 1 Division of Rheumatology, Hospital Universitario de Canarias, 38320 La Laguna, Spain; antonioazes87@gmail.com (A.A.-E.); margagon23@hotmail.com (M.G.-G.); martahediaz@gmail.com (M.H.-D.); 2 Division of Central Laboratory, Hospital Universitario de Canarias, 38320 La Laguna, Spain; fuensanta95@gmail.com (F.G.-B.); adeverag@gmail.com (A.d.V.-G.); alejandra.gd88@gmail.com (A.G.-D.); 3 Division of Rheumatology, IIS-Fundación Jiménez Díaz, 28040 Madrid, Spain; 3bheraselena@gmail.com; 4 Division of Rheumatology, Hospital Doctor Negrín, 35010 Las Palmas de Gran Canaria, Spain; adrian-ce@hotmail.es (A.Q.-R.); quevedojcarlos@yahoo.es (J.C.Q.-A.); 5 Division of Rheumatology, Hospital Universitario de La Princesa, IIS-Princesa, 28006 Madrid, Spain; scastas@gmail.com; 6 Department of Medicine and Psychiatry, University of Cantabria, 39005 Santander, Spain; 7 Department of Internal Medicine, University of La Laguna (ULL), 38200 La Laguna, Spain

**Keywords:** rheumatoid arthritis, METS-IR index, cardiovascular risk

## Abstract

**Background**: The Metabolic Score for Insulin Resistance (METS-IR) is a newly developed index that has been described to predict cardiovascular (CV) events. In this study, we calculated the METS-IR index in patients with rheumatoid arthritis (RA), a condition linked to an elevated CV risk. We then examined its relationship with disease characteristics and CV comorbidities, including disease activity, lipid profile, subclinical carotid atherosclerosis, and insulin resistance indices. **Methods:** A total of 515 RA patients were recruited. Disease-related characteristics and disease activity indices, including the Disease Activity Score (DAS28), the Clinical Disease Activity Index (CDAI), and the Simple Disease Activity Index (SDAI) were calculated. Additionally, the complete lipid profile, insulin resistance indices, metabolic syndrome criteria, and carotid ultrasound for intima–media thickness and carotid plaque detection were assessed. METS-IR was calculated. A multivariable linear regression analysis was performed to examine the associations between the disease characteristics and METS-IR. **Results:** METS-IR was positively correlated with age, body mass index, and traditional cardiovascular risk factors such as metabolic syndrome and insulin resistance indices. Carotid intima–media thickness—but not the presence of carotid plaque—was associated with significantly higher METS-IR values. Regarding disease-related characteristics, C-reactive protein and disease activity indices demonstrated a significant positive association with METS-IR after multivariable adjustment. Specifically, C-reactive protein was associated with higher METS-IR values (beta coefficient 0.2, 95% CI: 0.1–0.3, *p* < 0.001). All disease activity indices, except CDAI, showed a significant positive relationship with METS-IR. **Conclusions:** METS-IR is linked not only to CV risk factors but also, independently, to inflammatory disease activity in patients with RA. Its association with CV events in the general population and disease activity in RA highlights the significant role of inflammation in driving excessive cardiovascular risk in RA. This underscores the intricate relationship between metabolic dysfunction, systemic inflammation, and CV outcomes in RA.

## 1. Introduction

Rheumatoid arthritis (RA) is a chronic inflammatory disorder of uncertain etiology, primarily characterized by symmetric polyarthritis affecting peripheral joints. The disease, if left untreated, can progress to cartilage degradation and bone erosion, ultimately leading to joint destruction. While the synovium of diarthrodial joints is the primary site of pathology, RA can also affect various extra-articular organs and tissues, particularly in more severe cases. In this regard, non-articular manifestations of RA include systemic features such as cutaneous rheumatoid nodules, ocular issues like dryness, episcleritis, scleritis, parenchymal lung diseases, hematological abnormalities, and neurological manifestations [1]. Patients with RA exhibit an elevated risk of cardiovascular (CV) events, including myocardial infarction and CV-related mortality [2], compared to the general population [3]. Emerging research indicates that chronic inflammation in RA may accelerate the development of atherosclerosis. This association likely stems from mechanisms involving pro-inflammatory cytokines, immune complex deposition, and endothelial dysfunction. Dysregulated immune cells—including T cells, dendritic cells, and macrophages—play a central role by perpetuating systemic inflammation and driving the progression of atherosclerotic plaques. Intriguingly, overlapping inflammatory pathways appear to link synovial tissue (affected in RA) and vascular endothelium, suggesting shared molecular mechanisms between joint and vascular damage. Insulin resistance, characterized by the diminished responsiveness of cells to the glucose-lowering effects of insulin, is also prevalent in RA [4,5]. Furthermore, metabolic syndrome, which encompasses the simultaneous presence of metabolic risk factors associated with type 2 diabetes and CV disease, abdominal obesity, hyperglycemia, dyslipidemia, and hypertension, is frequently observed in patients with RA [6,7]. Metabolic syndrome has been described to increase the risk of CV disease in RA patients [8,9]. The diverse array of extra-articular manifestations, particularly the accelerated CV disease associated with RA, underscores the systemic nature of this condition and emphasizes the necessity for comprehensive patient evaluation and management.

The Metabolic Score for Insulin Resistance (METS-IR), introduced in 2018, is a novel index designed to assess cardiometabolic risk in both healthy and at-risk individuals, making it a promising tool for screening insulin sensitivity. METS-IR is calculated using the formula METS-IR = (Ln ((2 × glucose) + triglycerides) × BMI)/(Ln (HDL-cholesterol)). Therefore, it combines non-insulin fasting laboratory values and anthropometric measurements that can be easily obtained in a clinical setting. In its original description, it was found to have a good correlation with the euglycemic–hyperinsulinemic clamp, ectopic fat accumulation, and fasting insulin levels, which makes it a reliable indicator of overall insulin resistance [10]. Furthermore, in a cross-sectional analysis of NHANES data, each unit increase in METS-IR was associated with a 7% increase in the risk of type 2 diabetes [11]. Recent studies have found that METS-IR is associated with an increased risk of CV disease, stroke, and heart disease in middle-aged and elderly populations [12]. Specific cutoff values for the METS-IR may vary depending on the population being studied and the outcome of interest, and they have not yet been definitively established.

In this study, we calculated METS-IR in a large cohort of RA patients. We comprehensively assessed them by collecting not only disease-specific features but also measuring insulin resistance and subclinical carotid atheromatosis and generating a complete lipid profile. We then used multivariable analysis to explore how these characteristics relate to the METS-IR index. If any disease characteristics were associated with this index, it would underscore the importance of specific disease traits in the development of CV complications in RA patients. Therefore, the novelty of our study lies in its potential to elucidate the interplay between RA and metabolic health. Specifically, we aimed to investigate whether significant associations exist between the METS-IR and inflammation-related disease activity in RA patients. Such findings would deepen our understanding of the complex interplay between CV disease and inflammation in RA.

## 2. Materials and Methods

### 2.1. Study Participants

This was a cross-sectional study that included 515 patients with RA recruited consecutively. All of them were 18 years or older and fulfilled the 2010 ACR/EULAR classification criteria for RA (a set of guidelines used to classify RA in clinical practice and research) [13]. They had been diagnosed by rheumatologists and received regular follow-up appointments at rheumatology outpatient clinics. For inclusion in this study, the duration of RA had to be ≥1 year. As glucocorticoids are commonly used in RA treatment, patients taking prednisone or an equivalent dose of ≤10 mg/day were included. Exclusions were made for those with cancer, chronic diseases (e.g., hypothyroidism, heart or respiratory diseases, nephrotic syndrome), pregnancy, other inflammatory or autoimmune diseases (except secondary Sjögren’s syndrome), or active infections. The study was approved by the Institutional Review Committees at Hospital Universitario de Canarias and Hospital Universitario Doctor Negrín (approval number CHUC_2023_48, 25 May 2023), with all participants providing written informed consent. The research was conducted in compliance with relevant guidelines and the Declaration of Helsinki.

### 2.2. Data Collection

Participants underwent a thorough examination, including a CV risk factor and medication use questionnaire. The physical examination covered body mass index (BMI), abdominal circumference, and blood pressure (systolic and diastolic) measurements under standardized conditions. Information on smoking, diabetes, and hypertension was collected, and specific diagnoses and medication details were verified through medical record reviews. Disease activity in patients with RA was measured using the Disease Activity Score (DAS28) in 28 joints [14], the Clinical Disease Activity Index (CDAI) [15], and the Simple Disease Activity Index (SDAI) [16]. The presence of metabolic syndrome was determined using the National Cholesterol Education Program (NCEP/ATP III) criteria [17]. The NCEP ATP III definition states that metabolic syndrome is diagnosed if three or more of the following five criteria are present: waist circumference exceeding 102 cm in men or 88 cm in women, blood pressure above 130/85 mmHg, fasting triglyceride levels over 150 mg/dL, fasting high-density lipoprotein (HDL) cholesterol levels below 40 mg/dL in men or 50 mg/dL in women, and fasting blood glucose levels over 100 mg/d.

The Systematic Coronary Risk Evaluation-2 (SCORE2) CV risk tool was calculated as previously described using age, gender, smoking status, systolic blood pressure, and non-HDL-cholesterol [18]. SCORE2 estimates an individual’s 10-year risk of fatal and non-fatal CV disease events in individuals aged 40 to 69 years. For healthy people aged ⩾ 70 years, the SCORE2-OP (older persons) algorithm estimates 5-year and 10-year fatal and non-fatal CV disease events. METS-IR data at recruitment were calculated as described by Bello-Chavolla et al. [10]: METS-IR = (Ln ((2 × glucose) + triglycerides) × BMI)/(Ln (HDL-cholesterol)), where fasting glucose and triglyceride concentrations are used.

### 2.3. Laboratory Assessments

Serum lipid profiles were analyzed through enzyme-based colorimetric analysis (Roche Diagnostics, Basel, Switzerland) for total cholesterol, triglycerides, and HDL-C quantification. Lipoprotein subfractions were evaluated via immunoturbidimetric quantification (Roche Diagnostics). Measured concentrations spanned total cholesterol from 0.08 to 20.7 mmol/L, with an intra-assay coefficient of variation of 0.3%; triglycerides from 4 to 1000 mg/dL, with an intra-assay coefficient of variation of 1.8%; and HDL-C from 3 to 120 mg/dL, with an intra-assay coefficient of variation of 0.9%. Cardiovascular risk indices were derived as follows: the atherogenic index was calculated using the total cholesterol to HDL-C ratio according to Castelli’s equation, and LDL-C was calculated using Friedewald’s formula. Inflammatory markers were assessed using the erythrocyte sedimentation rate (ESR) determined by the standard Westergren methodology, and high-sensitivity CRP levels were measured using an ultra-sensitive immunometric assay. 

The homeostatic model assessment (HOMA) method was used to determine IR. The HOMA model, specifically HOMA2 in this study, estimates insulin sensitivity (%S) and β-cell function (%B) using fasting plasma insulin, C-peptide, and glucose levels. HOMA2, an updated computer-based version [19], assesses these functions from paired fasting glucose and either insulin or C-peptide concentrations, applicable within insulin ranges of 1–2200 pmol/L and glucose ranges of 1–25 mmol/L. C-peptide provides a more accurate measure of β-cell function as it directly reflects secretion, whereas insulin levels are preferred for calculating %S, which is derived from glucose disposal relative to insulin concentration. In our study, insulin serum levels were used to calculate insulin resistance (IR) and %S, while C-peptide serum levels determined %B. The HOMA2 model outputs insulin sensitivity as HOMA2-%S, where 100% represents normal sensitivity. HOMA2-IR, the insulin resistance index, is the inverse of %S. Insulin and C-peptide levels were measured using chemiluminescent immunometric assays (Architect Abbott, 2000I, Abbot Park, IL, USA for insulin and Immulite 2000, Siemens, Munich, Germany for C-peptide).

### 2.4. Carotid Ultrasound Assessment

A carotid ultrasound was performed to evaluate carotid intima–media thickness (cIMT) in the common carotid artery. This aimed to identify any localized plaques within the extracranial carotid arteries. Measurements were obtained using the Esaote MyLab 70 ultrasound system (Genoa, Italy) equipped with a 7–12 MHz linear transducer. The system employed the Quality Intima–Media Thickness (QIMT) real-time automated software-guided radiofrequency technique developed by Esaote in Maastricht, Netherlands. The assessment process followed the guidelines set in the Mannheim consensus [20], which establishes criteria for identifying plaques within the accessible extracranial carotid arteries. These arteries include the common carotid artery, the bulb, and the internal carotid artery. Plaque criteria were established as the presence of a localized bulge within the arterial lumen, with a measurement of cIMT exceeding >1.5 mm. Additionally, the bulge needed to be at least 50% larger than the adjacent cIMT or result in an arterial lumen reduction of >0.5 mm [20].

### 2.5. Statistical Analysis

Demographic and clinical characteristics of patients with RA were presented as means (standard deviation) for continuous variables and as percentages for categorical variables. For continuous variables with non-normal distribution, data were expressed as the median and interquartile range (IQR). The relationships between disease characteristics and METS-IR were analyzed using multivariable linear regression analysis. The multivariate relationship between disease characteristics and METS-IR values was adjusted for covariates associated with this index at a significance level of *p* < 0.20. To avoid collinearity, variables already included in the METS-IR formula were excluded from the multivariate adjustment. All analyses were conducted with a 5% two-sided significance level using Stata software, version 17/BE (StataCorp, College Station, TX, USA); *p*-values <0.05 were considered statistically significant.

## 3. Results

### 3.1. Demographics and Disease-Related Data

This study included 515 patients diagnosed with RA. The METS-IR values in our study were 42 ± 10. Demographic and disease-related characteristics of the participants are presented in Table 1. The mean age of the study population was 56 ± 10 years, with 81% of participants being women. The average BMI was 28 ± 5 kg/m^2^. The prevalence of classical CV risk factors was generally high in the study population. Specifically, 22% were current smokers at the time of enrollment, 14% had diabetes mellitus, and 34% were diagnosed with hypertension. Additionally, 54% met the diagnostic criteria for metabolic syndrome, and 32% and 11% of subjects were, respectively, on statin and aspirin therapy. The median value from the SCORE2 CV risk calculator was 3.9 (IQR 1.9–6.2), with 57% of RA patients having low risk, 28% of patients having intermediate risk, and 15% being at high risk. Regarding subclinical atherosclerosis markers, the mean intima–media thickness was 703 ± 141 microns and 46% of patients presented with carotid plaque. The full lipid profile and insulin resistance indices are shown in Table 1.

The median duration of the disease was 8 years (IQR 4–15). At the time of the study, the mean values of hs-CRP and ESR were 2.3 mg/l (IQR 1.0–5.4) and 17 mm/1st hour (IQR 7–32), respectively. Rheumatoid factor was positive in 75% of patients, and 69% were positive for anti-citrullinated protein antibodies (ACPAs). The disease activity, as assessed by DAS28-ESR, was 3.27 ± 1.38. The DAS28-CRP was 2.85 ± 1.14, while the SDAI and CDAI values were 12 (IQR 6–19) and 7 (IQR 3–14), respectively. Thirty-seven percent of the patients were treated with prednisone, and 88% were receiving at least one type of conventional DMARD, with methotrexate being the most commonly used (73%). Twenty percent of the patients were on anti-tumor necrosis factor therapies. The frequency of other treatments and historical disease-related data are detailed in Table 1.

### 3.2. Univariable Analysis of the Relationship Between METS-IR and Cardiovascular Traditional Risk Factors, Lipid Pattern, Insulin Resistance, and Carotid Subclinical Atherosclerosis

In general, the univariable relationship between various CV risk factors and METS-IR was strong and significant (Table 2). Age and body composition parameters, such as BMI and abdominal and hip circumferences, showed a positive and significant association with METS-IR. Conversely, female sex was associated with lower METS-IR values compared to male RA patients.

Regarding CV risk factors like obesity, hypertension, diabetes, and metabolic syndrome, patients exhibiting these conditions demonstrated significantly higher METS-IR values. Additionally, statin and aspirin intake correlated with elevated METS-IR levels. The CV risk calculator SCORE2, when considered both as a continuous and categorical variable, was associated with significantly higher METS-IR values (Table 2).

The lipid profile also exhibited numerous positive correlations with METS-IR. As expected, HDL cholesterol and triglycerides showed strong associations due to their inclusion in the METS-IR formula. However, despite this inherent relationship, non-HDL cholesterol levels correlated with significantly higher METS-IR values, while apolipoprotein A1 was associated with lower METS-IR levels (Table 2). This was also true for insulin resistance indices. Insulin levels, C-peptide, and markers of insulin resistance and beta-cell function were all significantly associated with higher METS-IR values in every case. Finally, cIMT showed a positive and significant correlation with METS-IR. However, this association was not observed for the presence of carotid plaque (Table 2).

### 3.3. Multivariable Analysis of the Association of Disease-Related Data with METS-IR

**Figure 1:**
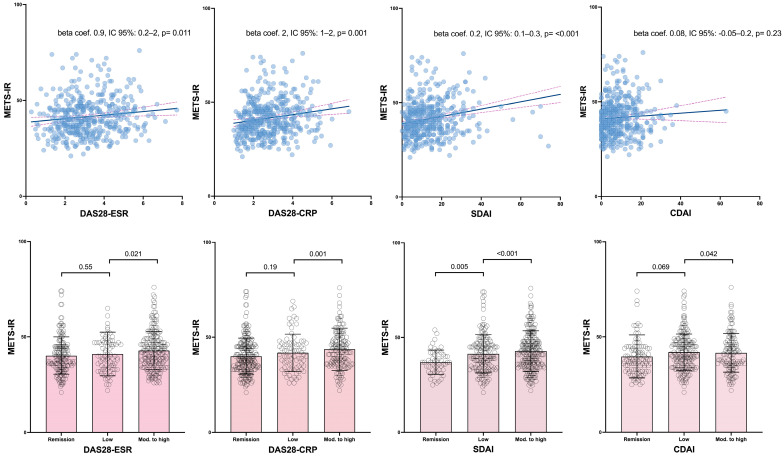
Relationship between continuous and categorized disease activity scores and the METS-IR index.

The results of the multivariate relationship between disease characteristics and METS-IR are presented in Table 3 and Figure 1. These analyses were adjusted for age, sex, hypertension, diabetes, and statin use. Disease duration and positivity for rheumatoid factor or ACPAs showed no significant association with METS-IR. However, CRP and disease activity indices showed a significant positive association with METS-IR. Specifically, CRP, after multivariate adjustment, was associated with higher METS-IR values (beta coefficient 0.2, 95% CI: 0.1–0.3, *p* < 0.001). When analyzed as continuous variables, all disease activity indices, except CDAI, demonstrated a significant positive correlation with METS-IR. Similarly, when disease activity indices were assessed categorically, moderate and high activity categories (except for CDAI) were associated with higher METS-IR values compared to the remission category, following multivariable adjustment (Table 3 and Figure 1).

The remaining disease characteristics, including extra-articular manifestations and the use of various therapies, showed no association with METS-IR, except for prednisone use. After adjusting for covariates, prednisone intake demonstrated a negative relationship with METS-IR (beta coefficient −2, 95% CI: −4 to −0.07, *p* = 0.043) (Table 3).

## 4. Discussion

Our study is the first in the literature to describe an association between RA’s disease activity and METS-IR values. Our findings demonstrate a link between disease activity and a CV risk index associated with CV events, suggesting that disease activity itself may independently elevate CV risk in patients with RA.

Although METS-IR only includes glucose, HDL cholesterol, triglycerides, and BMI, its association with all traditional cardiovascular risk factors was strong in our patients with RA. For example, it showed a significant positive relationship with age, male sex, hypertension, and diabetes. Additionally, despite not being based on plasma insulin levels, it demonstrated a significant and positive correlation with indices of insulin resistance and beta-cell dysfunction. Non-HDL, but not LDL, was associated with METS-IR. This finding is likely attributable to the fact that non-HDL cholesterol encompasses a broader spectrum of atherogenic lipoproteins, including very low-density lipoproteins (VLDLs) and intermediate-density lipoproteins (IDLs). These lipoproteins may be more closely associated with insulin resistance and metabolic dysfunction compared to LDL cholesterol alone. Furthermore, its association with SCORE2 was significant and noteworthy. This aligns with multiple reports highlighting the association of METS-IR with numerous CV disease features. For example, METS-IR has been shown to predict new-onset type 2 diabetes [21], to be associated with the risk of hypertension in the general adult population [22], and to correlate with most of the echocardiographic features of abnormal left ventricular geometry and function [23]. Therefore, we can conclude that this index maintains these relationships in patients with RA, despite this population typically exhibiting distinct insulin resistance [24] and inflammatory dyslipidemia [25] profiles compared to individuals without arthritis.

Although the METS-IR index was recently developed, some studies have explored its role in CV disease of patients with RA. In a retrospective cohort study involving 1218 RA patients from the National Health and Nutrition Examination Survey (NHANES 1999–2018), elevated METS-IR was linked to a significantly increased risk of CV disease mortality in RA patients [26]. Similarly, in a 7-year prospective cohort study (2011–2018) involving 1059 Chinese patients with RA, after adjusting for confounding factors, each quartile increase in METS-IR was linked to a 36% higher risk of CV disease [27]. In comparison to the lowest quartile, the highest quartile demonstrated a 63% increased risk. The authors concluded that METS-IR is an effective tool for predicting CV disease risk in Chinese patients with RA, offering new strategies for early identification and prevention of risk. However, none of these studies evaluated how METS-IR relates to disease characteristics. As a result, our study offers new insights into the effectiveness of this index in individuals with RA.

When examining the relationship between METS-IR and disease activity, we used four different activity scores. A significant association was observed for all scores except CDAI, which does not incorporate acute-phase reactants in its formula. METS-IR also showed a strong and significant correlation with CRP levels, suggesting that CRP may drive the relationship between METS-IR and indices such as SDAI and DAS28-CRP, which include CRP in their calculations. However, the association with DAS28-ESR persisted, despite this score not incorporating CRP. Additionally, while the univariable relationship between CDAI and METS-IR was not confirmed in multivariable analysis, a trend was observed. With respect to this, CDAI includes patient and physician global assessments, which are more subjective and may not always align with metabolic dysfunction. Our data indicate that the METS-IR score is strongly associated with systemic inflammation and insulin resistance, which are often reflected in CRP and ESR levels. Since CDAI relies solely on clinical measures (tender/swollen joint count and physician/patient assessments), it may not effectively capture the metabolic–inflammatory link. In contrast, inflammation-driven metabolic alterations are more objectively captured by laboratory-based indexes. For this reason, despite some discrepancies among the disease activity scores used, we believe our findings conclusively demonstrate a link between METS-IR and inflammatory disease activity.

We also observed a negative relationship between prednisone use and METS-IR. This negative association between prednisone use and METS-IR is somewhat surprising, as glucocorticoids are generally known to worsen metabolic profiles, including increasing insulin resistance, weight gain, and dyslipidemia. This unexpected finding suggests that prednisone use in our study population may not directly affect metabolic dysfunction but could instead be a marker of lower disease activity. Another possible explanation is that patients with more active RA and higher inflammation may be less likely to be on long-term prednisone, or they may receive lower doses as part of a well-managed treatment strategy. Furthermore, patients on stable prednisone regimens might have better-controlled disease, leading to reduced systemic inflammation and, consequently, a more favorable metabolic profile.

A strength of our study is the inclusion of a large patient cohort, allowing for a thorough multivariable analysis. However, we recognize the cross-sectional design of our study, which limits our ability to establish causality. Future prospective studies are needed to clarify the relationship between this score and CV event risk in RA patients, as well as how disease activity influences this association. Furthermore, since RA is a disease that primarily affects women, and the majority of our patients are female, we cannot conclude that the relationships found in our patients would differ in male subjects. For this reason, it will be necessary to validate our findings in male patients with RA.

## 5. Conclusions

In conclusion, the METS-IR demonstrates a significant correlation with CV risk factors in RA patients, mirroring observations in the general population. Furthermore, the METS-IR exhibits an independent association with disease activity and C-reactive protein (CRP) levels in RA patients. These findings highlight the potential utility of METS-IR as an important connection between CV risk and disease activity in RA patients. We believe that calculating METS-IR in routine clinical practice is straightforward and could provide valuable insights into disease activity, given its correlation with inflammatory activity, as well as the metabolic risk in patients with RA. Therefore, METS-IR may serve as a practical tool for integrating cardiometabolic risk assessment into RA management strategies.

## Figures and Tables

**Table 1:** Demographic information, cardiovascular risk factors, and disease-related data in RA patients.

	Rheumatoid Arthritis
	(*n* = 515)
METS-IR	42 ± 10
Age, years	56 ± 10
Female, *n* (%)	417 (81)
BMI, kg/m^2^	28 ± 5
Abdominal circumference, cm	96 ± 13
Hip circumference, cm	105 ± 11
Abdominal-to-hip ratio	1.14 ± 4.93
Cardiovascular data	
CV risk factors, *n* (%)	
Current smoker	112 (22)
Obesity	160 (34)
Hypertension	177 (34)
Diabetes Mellitus	70 (14)
Metabolic syndrome, *n* (%)	272 (56)
Statins, *n* (%)	166 (32)
Aspirin, *n* (%)	38 (11)
SCORE2, %	3.9 (1.9–6.2)
Low to moderate	292 (57)
High	144 (28)
Very high	79 (15)
Carotid ultrasound	
cIMT, microns	703 ± 141
Carotid plaque, *n* (%)	222 (46)
Analytical	
Total cholesterol, mg/dL	202 ± 39
Triglycerides, mg/dL	145 ± 83
HDL-cholesterol, mg/dL	56 ± 15
LDL-cholesterol, mg/dL	117 ± 34
LDL:HDL cholesterol ratio	2.22 ± 0.92
Non-HDL cholesterol, mg/dL	146 ± 39
Lipoprotein (a), mg/dL	33 (11–96)
Apolipoprotein A1, mg/dL	172 ± 32
Apolipoprotein B, mg/dL	106 ± 43
Apo B:Apo A ratio	0.63 ± 0.26
Insulin resistance indices	
Glucose, mg/dL	94 ± 23
Insulin, µU/mL	16 ± 26
C-peptide, ng/mL	3.42 ± 2.70
HOMA2-IR	1.17 (0.76–2.00)
HOMA2-S%	100 ± 71
HOMA2-B%-C-peptide	163 ± 76
Disease-related data	
Disease duration, years	8 (4–15)
CRP at time of study, mg/L	2.3 (1.0–5.4)
ESR at time of study, mm/1st hour	17 (7–32)
Rheumatoid factor, *n* (%)	377 (75)
ACPAs, *n* (%)	326 (69)
DAS28-ESR	3.27 ± 1.38
Remission	180 (36)
Low disease activity	89 (18)
Moderate disease activity	182 (36)
High disease activity	55 (11)
DAS28-CRP	2.85 ± 1.14
Remission	246 (49)
Low disease activity	86 (17)
Moderate disease activity	151 (30)
High disease activity	24 (5)
SDAI	12 (6–19)
Remission	53 (11)
Low disease activity	166 (35)
Moderate disease activity	197 (41)
High disease activity	63 (13)
CDAI	7 (3–14)
Remission	122 (24)
Low disease activity	212 (42)
Moderate disease activity	137 (27)
High disease activity	38 (7)
History of extraarticular manifestations, *n* (%)	65 (14)
Erosions, *n* (%)	195 (41)
Current drugs, *n* (%)	
Prednisone	193 (37)
Prednisone doses, mg/day	5.34 ± 3.67
NSAIDs	210 (41)
DMARDs	452 (88)
Methotrexate	378 (73)
Leflunomide	109 (21)
Hydroxychloroquine	54 (10)
Salazopyrin	31 (6)
Anti-TNF therapy	101 (20)
Tocilizumab	25 (5)
Rituximab	11 (2)
Abatacept	17 (3)
Baricitinib	8 (2)
Tofacitinib	5 (1)

Data represent means ± SD, or median (interquartile range) when data were not normally distributed. CV: cardiovascular; LDL: low-density lipoprotein; HDL: high-density lipoprotein; CRP: C-reactive protein; NSAIDs: Nonsteroidal anti-inflammatory drug; DMARD: disease-modifying antirheumatic drug; TNF: tumor necrosis factor; ESR: erythrocyte sedimentation rate; BMI: body mass index; DAS28: Disease Activity Score in 28 joints; ACPAs: Anti-citrullinated protein antibodies; HOMA: homeostatic model assessment; CDAI: Clinical Disease Activity Index; SDAI: Simple Disease Activity Index; cIMT: carotid intima–media thickness.

**Table 2:** Univariable association between cardiovascular disease-related data and METS-IR index.

	METS-IR	
	Beta Coefficient (95% CI), *p*	
Age, years	**0.1 (0.03–0.2)**	**0.007**
Female, *n* (%)	**−4 (−1–6)**	**0.002**
BMI, kg/m^2^	**2 (2–2)**	**<0.001**
Abdominal circumference, cm	**1 (1–1)**	**<0.001**
Hip circumference, cm	**1 (1–1)**	**<0.001**
Abdominal-to-hip ratio	0.2 (−0.1–0.5)	0.31
Cardiovascular data		
CV risk factors, *n* (%)		
Current smoker	**−4 (−6–(−1))**	**0.002**
Obesity	**15 (14–17)**	**<0.001**
Hypertension	**5 (3–7)**	**<0.001**
Diabetes Mellitus	**5 (2–7)**	**0.001**
Metabolic syndrome	**9 (7–11)**	**<0.001**
Statins, *n* (%)	**3 (1–5)**	**0.001**
Aspirin, *n* (%)	**4 (0–8)**	**0.040**
SCORE2, %	**0.5 (0.2–0.7)**	**<0.001**
Low to moderate	**ref.**	
High	**3 (0.5–5)**	**0.015**
Very high	**3 (0.02–6)**	**0.048**
Analytical		
Total cholesterol, mg/dL	−0.01 (−0.03–0.01)	0.44
Triglycerides, mg/dL	**0.06 (0.05–0.07)**	**<0.001**
HDL-cholesterol, mg/dL	**−0.3 (−0.4–−0.3)**	**<0.001**
LDL-cholesterol, mg/dL	−0.01 (−0.04–0.02)	0.38
LDL:HDL cholesterol ratio	**4 (3–5)**	**<0.001**
Non-HDL cholesterol, mg/dL	**0.05 (0.02–0.07)**	**<0.001**
Lipoprotein (a), mg/dL	0.01 (−0.01–0.02)	0.29
Apolipoprotein A1, mg/dL	**−0.1 (−0.2–−0.1)**	**<0.001**
Apolipoprotein B, mg/dL	0.01 (−0.01–0.03)	0.32
Apo B:Apo A1 ratio	**14 (11–17)**	**<0.001**
Insulin resistance indices		
Glucose, mg/dL	**0.1 (0.06–0.14)**	**<0.001**
Insulin, µU/mL	**0.11 (0.08–0.15)**	**<0.001**
C-peptide, ng/mL	**1 (1–2)**	**<0.001**
HOMA2-IR	**2 (2–3)**	**<0.001**
HOMA2-S%	**−0.06 (−0.07–−0.05)**	**<0.001**
HOMA2-B%-C-peptide	**0.04 (0.02–0.05)**	**<0.001**
Carotid ultrasound		
cIMT, microns	**0.01 (0.002–0.02)**	**0.01**
Carotid plaque, *n* (%)	0.6 (−1.3–2.5)	0.54

In this analysis, the METS-IR index is the dependent variable. Significant *p* values are depicted in bold. CV: cardiovascular; LDL: low-density lipoprotein; HDL: high-density lipoprotein; BMI: body mass index; HOMA: homeostatic model assessment; cIMT: carotid intima–media thickness. SCORE2: Systematic Coronary Risk Evaluation 2; METS-IR: Metabolic Score for Insulin Resistance.

**Table 3:** Association between disease-related data and METS-IR.

	METS-IR			
	Univariable		Multivariable	
	Beta Coefficient (95% Confidence Interval), *p*			
Disease duration, years	−0.008 (−0.1–0.1)	0.88		
CRP at time of study, mg/L	**0.2 (0.2–0.3)**	**<0.001**	**0.2 (0.1–0.3)**	**<0.001**
ESR at time of study, mm/1° hour	0.04 (−0.01–0.09)	0.15	0.03 (−0.02–0.08)	0.29
Rheumatoid factor, *n* (%)	−1 (−4–0.8)	0.23		
ACPAs, *n* (%)	−2 (−4–0.3)	0.090	−0.8 (−3–1)	0.43
DAS28-ESR	**0.9 (0.2–2)**	**0.011**	**0.8 (0.09–2)**	**0.028**
Remission	ref.		ref.	
Low disease activity	1 (−2–4)	0.55	0.9 (−2–4)	0.50
Moderate and high disease activity	**3 (0.4–5)**	**0.021**	**2 (0.09–4)**	**0.041**
DAS28-PCR	**2 (1–2)**	**0.001**	**1 (0.5–2)**	**0.002**
Remission	ref.		ref.	
Low disease activity	2 (−1–4)	0.19	2 (−1–4)	0.24
Moderate and high disease activity	**4 (1–6)**	**0.001**	**3 (1–5)**	**0.002**
SDAI	**0.2 (0.1–0.3)**	**<0.001**	**0.2 (0.1–0.2)**	**<0.001**
Remission	ref.		ref.	
Low disease activity	**5 (1–8)**	**0.005**	**3 (0.3–6)**	**0.030**
Moderate and high disease activity	**6 (3–9)**	**<0.001**	**5 (2–8)**	**0.002**
CDAI	0.08 (−0.05–0.2)	0.23	0.07 (−0.06–0.2)	0.28
Remission	ref.		ref.	
Low disease activity	2 (−0.2–5)	0.069	1 (−1–4)	0.32
Moderate and high disease activity	**3 (0.09–5)**	**0.042**	2 (−0.6–4)	0.14
History of extraarticular manifestations, *n* (%)	−0.3 (−3–3)	0.86		
Erosions, *n* (%)	−2 (−4–0.3)	0.089	−2 (−4–0.2)	0.074
Current drugs, *n* (%)				
Prednisone	−2 (−4–0.004)	0.050	**−2 (−4–(−0.07))**	**0.043**
Prednisone doses, mg/day	−0.1 (−0.4–0.3)	0.72		
NSAIDs	0.4 (−1.5–2.3)	0.69		
DMARDs	−1 (−3–2)	0.73		
Methotrexate	−0.2 (−2.3–1.9)	0.84		
Leflunomide	−0.6 (−2.9–1.6)	0.58		
Hydroxychloroquine	−0.4 (−3.4–2.6)	0.80		
Salazopyrin	−2 (−6–2)	0.38		
Anti-TNF therapy	−0.1 (−2.5–2.3)	0.92		
Tocilizumab	0.1 (−4.2–4.4)	0.98		
Rituximab	−0.1 (−6.2–6)	0.98		
Abatacept	0.2 (−4.9–5.3)	0.94		
Baricitinib	1 (−6–9)	0.72		
Tofacitinib	−1 (−8–6)	0.73		

In this analysis, the METS-IR index is the dependent variable. Significant *p* values are depicted in bold. The multivariable analysis is adjusted for age, sex, smoking, hypertension, diabetes, and the use of statins. NSAIDs: Nonsteroidal anti-inflammatory drugs; DMARD: disease-modifying antirheumatic drug; TNF: tumor necrosis factor; ESR: erythrocyte sedimentation rate; DAS28: Disease Activity Score in 28 joints; CRP: C-reactive protein; ACPAs: Anti-citrullinated protein antibodies; CDAI: Clinical Disease Activity Index; SDAI: Simple Disease Activity Index; METS-IR: Metabolic Score for Insulin Resistance.

## Data Availability

Data will be made available on request.

## Abbreviations

The following abbreviations are used in this manuscript:

METS-IR	Metabolic Score for Insulin Resistance
CV	Cardiovascular
RA	Rheumatoid arthritis
DAS28	Disease Activity Score
CDAI	Clinical Disease Activity Index
SDAI	Simple Disease Activity Index
CI	Confidence interval
SCORE2	Systematic Coronary Risk Evaluation-2
HDL	High-density lipoprotein
LDL	Low-density lipoprotein
CRP	C-reactive protein
HOMA	Homeostatic model assessment
cIMT	Carotid intima–media wall
IQR	Interquartile range
BMI	Body mass index
ESR	Erythrocyte sedimentation rate
ACPAs	Anti-citrullinated protein antibodies
NSAIDs	Nonsteroidal anti-inflammatory drugs
DMARDs	Disease-modifying antirheumatic drug
TNF	Tumor necrosis factor

## References

[1] Conforti A., Di Cola I., Pavlych V., Ruscitti P., Berardicurti O., Ursini F., Giacomelli R., Cipriani P. (2021). Beyond the Joints, the Extra-Articular Manifestations in Rheumatoid Arthritis. Autoimmun. Rev..

[2] Wolfe F., Michaud K. (2008). The Risk of Myocardial Infarction and Pharmacologic and Nonpharmacologic Myocardial Infarction Predictors in Rheumatoid Arthritis: A Cohort and Nested Case-Control Analysis. Arthritis Rheum..

[3] Aviña-Zubieta J.A., Choi H.K., Sadatsafavi M., Etminan M., Esdaile J.M., Lacaille D. (2008). Risk of Cardiovascular Mortality in Patients with Rheumatoid Arthritis: A Meta-Analysis of Observational Studies. Arthritis Rheum..

[4] Quevedo-Abeledo J.C., Sánchez-Pérez H., Tejera-Segura B., de Armas-Rillo L., Ojeda S., Erausquin C., González-Gay M., Ferraz-Amaro I. (2021). Higher Prevalence and Degree of Insulin Resistance in Patients with Rheumatoid Arthritis than in Patients with Systemic Lupus Erythematosus. J. Rheumatol..

[5] Tejera-Segura B., López-Mejías R., De Vera-González A.M., Jiménez-Sosa A., Olmos J.M., Hernández J.L., Llorca J., González-Gay M.A., Ferraz-Amaro I. (2019). Relationship between Insulin Sensitivity and β-Cell Secretion in Nondiabetic Subjects with Rheumatoid Arthritis. J. Rheumatol..

[6] Ferraz-Amaro I., González-Juanatey C., López-Mejias R., Riancho-Zarrabeitia L., González-Gay M.A. (2013). Metabolic Syndrome in Rheumatoid Arthritis. Mediat. Inflamm..

[7] Cai W., Tang X., Pang M. (2022). Prevalence of Metabolic Syndrome in Patients with Rheumatoid Arthritis: An Updated Systematic Review and Meta-Analysis. Front. Med..

[8] Santos-Moreno P., Rodríguez-Vargas G.-S., Martínez S., Ibatá L., Rojas-Villarraga A. (2022). Metabolic Abnormalities, Cardiovascular Disease, and Metabolic Syndrome in Adult Rheumatoid Arthritis Patients: Current Perspectives and Clinical Implications. Open Access Rheumatol..

[9] Castañeda S., Nurmohamed M.T., González-Gay M.A. (2016). Cardiovascular Disease in Inflammatory Rheumatic Diseases. Best Pr. Res. Clin. Rheumatol..

[10] Bello-Chavolla O.Y., Almeda-Valdes P., Gomez-Velasco D., Viveros-Ruiz T., Cruz-Bautista I., Romo-Romo A., Sánchez-Lázaro D., Meza-Oviedo D., Vargas-Vázquez A., Campos O.A. (2018). METS-IR, a Novel Score to Evaluate Insulin Sensitivity, Is Predictive of Visceral Adiposity and Incident Type 2 Diabetes. Eur. J. Endocrinol..

[11] Hou Y., Li R., Xu Z., Chen W., Li Z., Jiang W., Meng Y., Han J. (2024). Association of METS-IR Index with Type 2 Diabetes: A Cross-Sectional Analysis of National Health and Nutrition Examination Survey Data from 2009 to 2018. PLoS ONE.

[12] Qian T., Sheng X., Shen P., Fang Y., Deng Y., Zou G. (2023). Mets-IR as a Predictor of Cardiovascular Events in the Middle-Aged and Elderly Population and Mediator Role of Blood Lipids. Front. Endocrinol..

[13] Aletaha D., Neogi T., Silman A.J., Funovits J., Felson D.T., Bingham C.O., Birnbaum N.S., Burmester G.R., Bykerk V.P., Cohen M.D. (2010). 2010 Rheumatoid Arthritis Classification Criteria: An American College of Rheumatology/European League Against Rheumatism Collaborative Initiative. Ann. Rheum. Dis..

[14] Prevoo M.L.L., Van’T Hof M.A., Kuper H.H., Van Leeuwen M.A., Van De Putte L.B.A., Van Riel P.L.C.M. (1995). Modified Disease Activity Scores That Include Twenty-eight-joint Counts Development and Validation in a Prospective Longitudinal Study of Patients with Rheumatoid Arthritis. Arthritis Rheum..

[15] Smolen J.S., Breedveld F.C., Schiff M.H., Kalden J.R., Emery P., Eberl G., van Riel P.L., Tugwell P. (2003). A Simplified Disease Activity Index for Rheumatoid Arthritis for Use in Clinical Practice. Rheumatology.

[16] Aletaha D., Smolen J. (2005). The Simplified Disease Activity Index (SDAI) and the Clinical Disease Activity Index (CDAI): A Review of Their Usefulness and Validity in Rheumatoid Arthritis. Clin. Exp. Rheumatol..

[17] (2001). Expert Panel on Detection, Evaluation, and Treatment of High Blood Cholesterol in Adults. Executive Summary of the Third Report of the National Cholesterol Education Program (NCEP) Expert Panel on Detection, Evaluation, and Treatment of High Blood Cholesterol in Adults (Adult Treatment Panel III). JAMA.

[18] SCORE2 Working Group and ESC Cardiovascular Risk Collaboration (2021). SCORE2 Risk Prediction Algorithms: New Models to Estimate 10-Year Risk of Cardiovascular Disease in Europe. Eur. Heart J..

[19] Wallace T.M., Levy J.C., Matthews D.R. (2004). Use and Abuse of HOMA Modeling. Diabetes Care.

[20] Touboul P.J., Hennerici M.G., Meairs S., Adams H., Amarenco P., Bornstein N., Csiba L., Desvarieux M., Ebrahim S., Hernandez Hernandez R. (2012). Mannheim Carotid Intima-Media Thickness and Plaque Consensus (2004-2006-2011). An Update on Behalf of the Advisory Board of the 3rd, 4th and 5th Watching the Risk Symposia, at the 13th, 15th and 20th European Stroke Conferences, Mannheim, Germany, 2004, Brussels, Belgium, 2006, and Hamburg, Germany, 2011. Cerebrovasc. Dis..

[21] Cheng H., Jia Z., Li Y.T., Yu X., Wang J.J., Xie Y.J., Hernandez J., Wang H.H.X. (2024). Metabolic Score for Insulin Resistance and New-Onset Type 2 Diabetes in a Middle-Aged and Older Adult Population: Nationwide Prospective Cohort Study and Implications for Primary Care. JMIR Public Health Surveill..

[22] Rao K., Yang J., Wu M., Zhang H., Zhao X., Dong Y. (2023). Association Between the Metabolic Score for Insulin Resistance and Hypertension in Adults: A Meta-Analysis. Horm. Metab. Res..

[23] Ezhova N.E., Shavarova E.K., Kobalava Z.D., Bazdyreva E.I., Shavarov A.A. (2024). Metabolic Score for Insulin Resistance (METS-IR) Associations with Subclinical Left Ventricular and Left Atrial Remodelling in Young Subjects with Hypertension. Ann. Clin. Cardiol..

[24] Ferraz-Amaro I., García-Dopico J.A., Medina-Vega L., González-Gay M.A., Díaz-González F. (2013). Impaired Beta Cell Function Is Present in Nondiabetic Rheumatoid Arthritis Patients. Arthritis Res. Ther..

[25] Tejera-Segura B., Macía-Díaz M., Machado J.D., de Vera-González A., García-Dopico J.A., Olmos J.M., Hernández J.L., Díaz-González F., González-Gay M.A., Ferraz-Amaro I. (2017). HDL Cholesterol Efflux Capacity in Rheumatoid Arthritis Patients: Contributing Factors and Relationship with Subclinical Atherosclerosis. Arthritis Res. Ther..

[26] Zhou Y., Gao J. (2024). Association between Metabolic Score for Insulin Resistance and Cardiovascular Disease Mortality in Patients with Rheumatoid Arthritis: Evidence from the NHANES 1999–2018. Front. Endocrinol..

[27] Ke W., Xu L., Luo N. (2025). Predictive Value of Insulin Resistance Metabolic Score for Cardiovascular Disease in Chinese Arthritis Patients: A Prospective Cohort Study. Rheumatology.

